# Plasma level of LDL-cholesterol at diagnosis is a predictor factor of breast tumor progression

**DOI:** 10.1186/1471-2407-14-132

**Published:** 2014-02-26

**Authors:** Catarina Rodrigues dos Santos, Isabel Fonseca, Sérgio Dias, JC Mendes de Almeida

**Affiliations:** 1 Gulbenkian Programme for Advanced Medical Education, Lisbon, Portugal; 2 Department of Surgical Oncology, Instituto Português de Oncologia de Lisboa, Francisco Gentil, Lisbon, Portugal; 3 Department of Pathology, Instituto Português de Oncologia de Lisboa, Francisco Gentil, Lisbon, Portugal; 4 Instituto de Medicina Molecular, Lisbon, Portugal; 5 Faculdade de Medicina de Lisboa, Lisbon, Portugal

**Keywords:** LDL-cholesterol, Breast cancer, Tumor progression

## Abstract

**Background:**

Among women, breast cancer (BC) is the leading cancer and the most common cause of cancer-related death between 30 and 69 years. Although lifestyle and diet are considered to have a role in global BC incidence pattern, the specific influence of dyslipidemia in BC onset and progression is not yet completely understood.

**Methods:**

Fasting lipid profile (total cholesterol, LDL-C, HDL-C, and triglycerides) was prospectively assessed in 244 women with BC who were enrolled according to pre-set inclusion criteria: diagnosis of non-hereditary invasive ductal carcinoma; selection for surgery as first treatment, and no history of treatment with lipid-lowering or anti-diabetic drugs in the previous year. Pathological and clinical follow-up data were recorded for further inclusion in the statistical analysis.

**Results:**

Univariate associations show that BC patients with higher levels of LDL-C at diagnosis have tumors that are larger, with higher differentiation grade, higher proliferative rate (assessed by Ki67 immunostaining), are more frequently Her2-neu positive and are diagnosed in more advanced stages. Cox regression model for disease-free survival (DFS), adjusted to tumor T and N stages of TNM classification, and immunohistochemical subtypes, revealed that high LDL-C at diagnosis is associated with poor DFS. At 25 months of follow up, DFS is 12% higher in BC patients within the third LDL-C tertile compared to those in the first tertile.

**Conclusions:**

This is a prospective study where LDL-C levels, at diagnosis, emerge as a prognostic factor; and this parameter can be useful in the identification and follow-up of high-risk groups. Our results further support a possible role for systemic cholesterol in BC progression and show that cholesterol metabolism may be an important therapeutic target in BC patients.

## Background

Cancer and cardiovascular diseases are the leading causes of death in Europe [1] and USA [2] and their incidence is increasing also in Asia [3,4].

In what concerns cancer, breast cancer (BC) is the most frequent diagnosed each year in Europe and USA (age–adjusted incidence rate, 76–89,7 per 100,000) [5] and the most common cause of cancer-related death between 30 and 69 years [4]. In Asia, incidence rate of BC is lower (age–adjusted incidence rate, 22–30 per 100,000) [5] but is dramatically raising [4,6].

Lifestyle and diet are frequently indicated as reasons for the global distribution of BC incidence. Nevertheless, while dyslipidemia [high LDL-C (low density lipoprotein cholesterol) and low HDL-C (high density lipoprotein cholesterol) levels] was already shown to play a major role in the etiopathogenesis of cardiovascular diseases [7], mainly attributed to diet, the specific influence of dyslipidemia in BC initiation and progression is not completely understood.

Cholesterol is a structural component of the cell membrane, specially localized in lipid rafts - membrane microdomains that assemble the signal transduction machinery and associate to proteins implicated in key cellular signaling pathways. Many of these pathways closely associate with malignant transformation due to their influence in organization of the cytoskeleton, cell polarity and angiogenesis [8,9].

Furthermore, cholesterol is also a steroid hormone precursor and the vast majority of BC is known to be hormone responsive [10]. The peak incidence of BC occurs in the perimenopausal age [11], when women dyslipidemia prevalence also rises [12].

Several authors have shown that lipoprotein fractions can induce cancer cells proliferation and migration *in vitro*[13-19] and oxysterol 27-hydroxycholesterol, a primary metabolite of cholesterol was shown to promote ER–positive BC growth in *in vivo* models [20]. Moreover, studies in genetic or diet induced hypercholesterolemic mouse models also demonstrated a clear association between high lipid levels and BC development [21] and progression [22,23].

However, clinical data have provided contradictory results. Prospective studies showed positive association between total cholesterol (TC) levels and both BC incidence [24-26] and increased overall mortality in BC patients [27]; but also no association at all [28-33] or even inverse associations between TC levels and incidence of premenopausal BC [34-36]; as well as a protective effect of very high levels of TC [37].

LDL-C and HDL-C are lipoproteins responsible for the cholesterol transportation, LDL-C from the liver to peripheral tissues and HDL-C for the reverse transportation [38]. Studies specifically addressing the relation of lipoproteins fractions are similarly contradictory. Regarding HDL-C, some show a positive association between low HDL-C levels and increased BC risk [29,39-42] and a protective effect of high HDL to premenopausal BC [43]; while others find no association [44-46], and some even report a positive correlation between high HDL-C and BC risk [47,48]. LDL-C is less studied and no association with BC risk was reported [29,31]. Triglycerides levels were no longer associated with risk in prospective studies except, when in combination with low HDL [33,43].

Different study designs, study populations and endpoints, duration of follow up, timing of cholesterol measurements, tumor stage and histological type, and differences in statistical adjustment for confounding variables may account for the disparity in the results of these studies.

Although the inconsistency of the studies in the influence of plasma cholesterol on BC risk, alterations in lipid profile, are often seen among BC patients, when compared to non-cancer controls. Increased TC levels are transversal to all studies [41,49-54] with the exception to advanced cases in two studies [55,56]. Triglycerides and LDL, when measured, were also constantly raised while HDL level was decreased [41,49-54,57].

So, whereas from the epidemiological point of view, the influence of plasma cholesterol in BC initiation has been difficult to demonstrate, the variations of lipid profile in BC patients and controlled experimental studies suggest a role of cholesterol in BC progression.

In the present study we hypothesized that the host cholesterol-enriched macroenvironment, promotes breast tumor progression. To answer this question we prospectively assessed the lipid profile in a cohort of women with BC, at diagnosis, and correlated these levels with clinical and pathological data collected thereafter.

## Methods

### Study population and data collection

From January to December 2011, women, who underwent for operable BC at the Breast Unit of Instituto Português de Oncologia de Lisboa, Francisco Gentil (IPOLFG), were prospectively assembled.

Women were included if they met the following criteria: 1) invasive ductal carcinoma (currently named invasive carcinoma NOS [58]), confirmed by biopsy; 2) surgery as the first treatment; 3) informed consent. Women were excluded if they had: 1) previous treatment (chemotherapy, radiotherapy, hormonotherapy); 2) hereditary BC (confirmed by genetic analysis) or 3) were taking lipid-lowering, anti-diabetic drugs (statins, fibrates, oral anti diabetics, Insulin) or corticosteroids in the previous year. The study was approved by the Ethics Committee of the IPOLFG.

Demography, risk factors [menopausal status, body mass index (BMI), age, family history, parity, breast feeding] and clinical examination were recorded in the first interview.

Treatment was determined by the clinicopathological stage and patient characteristics according to the institutional protocols (following NCCN guidelines [59]), without changes related to the study.

Follow up, after surgery and adjuvant treatment (when appropriate), was scheduled every 6 months for 2 years and annual thereafter. Mammography was performed 1 year after surgery and then repeated yearly.

### Biospecimen collection and plasma lipid and lipoproteins assays

Fasting lipid profile was measured at diagnosis, along with routine preoperative exams. Blood was collected into EDTA-coated tubes and the plasma levels of total cholesterol (TC), low density lipoprotein (LDL-C), high density lipoprotein (HDL-C) and triglycerides were measured automatically by electrophoresis (Architect ci8200 analyzer; Abbott Diagnostics, Wiesbaden, Germany) at the certified Clinical Pathology Laboratory of IPOLFG.

### Pathology and Immunohistochemistry

Hormonal receptors were measured using standardized immunohistochemistry. Her2-neu receptor was scored according to the World Health Organization guidelines [60] from 0 to 3+. All cases with 2+ score were reevaluated using chromogenic *in situ* hybridation. Immunohistochemical staining for Ki 67 was performed in a Dako Autostainer® (Dako, Glostrup, Denmark) using standard protocols, followed by counting positive cells in an automated cellular imaging system (ACIS® II, Dako, Glostrup, Denmark I). Technicians were blinded to the lipid profile status of the study participants.

### Statistical analysis

Continuous variables are presented as mean (standard deviation) or median (interquartile range) if they have normal distribution or not, respectively. For categorical variables absolute values and frequencies are shown.

Spearman rank correlations coefficients were calculated to examine correlations between continuous variables.

Univariate analysis between lipid profile, BC risk factors [status, body mass index (BMI), age, family history, parity, breast feeding] and traditional prognostic factors [tumor size, positive lymph nodes, tumor grade, lymphovascular invasion (LVI), estrogen receptor (ER), progesterone receptor (PR), Her2-neu receptor (HER2) and Ki 67] were performed using parametric tests to variables with normal distribution and non-parametric tests to variables without normal distribution.

Multivariate logistic regression to the risk of tumor T stage included the following variables: TC (by tertiles), LDL (by tertiles), Triglycerides (3rd tertile), BMI (by tertiles) and age (by tertiles) by using stepwise conditional forward analysis method (entry 0,05; removal 0,10).

Kaplan-Meier curves were used to determine overall survival (OS) and disease-free survival (DFS) rates with use of log rank tests. Cox proportional hazards models were used to estimate hazard ratios with 95% confidence intervals (CI), relating LDL level to DFS. Multivariate Cox model was adjusted to tumor T stage, N stage and subtype.

To assess the internal validity of our results we examined the association of lipid profile with BMI and age. The association of BMI and tumor characteristics, as well as OS and DFS adjusted to BMI were also determined.

For statistical purposes, cases were censored at the date of disease progression confirmation, death or at June 9th, 2013, whichever came first.

Likelihood ratio *P* values are reported to whole variables in the model. All *P* values are two-tailed.

The statistical analysis was done using IBM SPSS Statistics for Windows, Version 19.0(Armonk, NY: IBM Corp. Released 2010).

## Results

A total of 446 women were potential assembled to the study. Of those, 202 were excluded for being on conflicting medications (n = 134), for BC histological type (n = 60) and previous treatments (n = 9). Baseline demographic, clinical and tumor related characteristics of the study population (n = 244) are listed in Table 1.

**Table 1:** Clinical and tumor-related characteristics of the study population (N = 244)

**Characteristics**		**No. of patients**	**%**^**1**^
**Patient characteristics**			
** Age (Years), mean±SD (range)**	58,2±13,3 (29-91)		
** Weight (Kg), median (interquartile range)**	67 (60-76)		
** Height (cm), median (interquartile range)**	160 (154-163)		
** BMI (Kg/m**^**2**^**), median (interquartile range)**	26,7 (23,5-30,44)		
** Menopausal status (yes)**		126	65,6
** Gestation (yes)**		182	90,54
** Breast-feeding (yes)**		146	79,3
** Oral contraception/HT (yes)**		96	55,6
** Family history of BC♯ (yes)**		56	27,5
** TC (mg/dL), median (interquartile range)**	209,5 (191-231)		
** HDL-C (mg/dL), median (interquartile range)**	53 (46-60)		
** LDL-C (mg/dL), median (interquartile range)**	128 (110-153)		
** Triglycerides (mg/dL), median (interquartile range)**	94 (74,5-126)		
**Tumor characteristics**^**2**^			
** Histological type**	IDC	244	100
** Tumor size (mm), median (interquartile range)**	21 (14-30)		
** Tumor stage (T)**	**T1, ≤ 2 cm**	122	50
	**T2, 2-5 cm**	115	47,2
	**T3, >5 cm**	7	2,8
** Tumor differentiation grade**	**G1**	27	12,4
	**G2**	134	61,5
	**G3**	57	26,2
** Immunohistochemical subtypes**	**ER/PR positive**	204	83,9
	**Triple negative**	23	9,5
	**HER 2 Type**	16	6,6
** LVI positive**		61	29,2
** Nodal stage (N)**	**N0**	137	56,9
	**N1, 1-3 LN+**	72	29,9
	**N2, 4-9 LN+**	18	7,47
	**N3, ≥10 LN+**	14	5,8
** Clinical stage**	**I**	84	34,4
	**II**	125	51,3
	**III**	35	14,3
** Bilaterality (yes)**		10	4,1

¹Frequency of known cases.

^2^Tumor characteristics based on The American Joint Committee on Cancer [58].

Histological type of breast cancer (IDC is presently named invasive carcinoma, NOS); Tumor Stage T of TNM Classification (T1≤2cm; T2:2-5cm; T3>5 cm); Tumor Differentiation Grade of Nothingham Histologic Score (G1, G2, G3); Immunohistochemical subtypes [Luminal A and B (ER/PR positive); Triple negative (ER/PR negative and Her2-neu negative); HER2 Type (ER/PR negative and Her2-neu positive)]; Pathological axillar Nodal Stage of TNM Classification (N0: no metastatic LN; N1: 1-3 metastatic LN; N2:4-9 metastatic lymph nodes; N3≥10 metastatic lymph nodes); Clinical Stage or Prognostic Stages Groups of American Joint Committee on Cancer (I, II, III,IV).

HT: Hormonal Therapy; BC: Breast Cancer; BMI: Body Mass Index; HT: Hormonal Therapy; TC: Total Cholesterol; LDL-C: Low Density Lipoprotein; HDL-C: High Density Lipoprotein IDC: Invasive Ductal Carcinoma; ER: Estrogen Receptor; PR: Progesterone Receptor; LVI: Lymphovascular Invasion; LN: Lymph Nodes.

All women underwent surgery, being the majority treated with breast-conserving surgery (73,8%); 78,3% had adjuvant radiotherapy; 67,7% had systemic chemotherapy; 11,9% are under trastuzumab and 70,1% are under hormone therapy (Additional file 1).

During follow up, 1 woman had local disease relapse, 16 women had systemic dissemination and 9 died (7 from confirmed disease progression) (Additional file 2).

### Spearman correlations

Exploratory correlations between lipid profile, age, BMI, primary tumor size and lymph node metastasis ratio (defined as the number of metastatic axillary lymph nodes, over the total lymph nodes removed), showed that systemic levels of LDL-C and TC correlates positively with tumor size (Spearman r = 0,199, *P* 0,002; Spearman r = 0,145, *P* 0,025, respectively). As expected, age correlates with BMI (Spearman r = 0,155, *P* 0,022) and triglycerides (Spearman r = 0,312, *P* <0, 0001) and BMI correlates with age, LDL-C (Spearman r = 0,161, *P* 0,018), HDL (Spearman r = −0,157, *P* 0,021) and triglycerides (Spearman r = 0,149, *P* 0,027).

Plasma LDL-C level was significantly related to tumor T stage and prognostic groups of the American Joint Committee on Cancer [58]. There was no statistical difference in other parameters of lipid profile across tumor stages (Additional file 3) and we did not find correlations between pathological or clinical data and lipid profile variables (Additional file 4).

### Univariate associations

**Figure 1:**
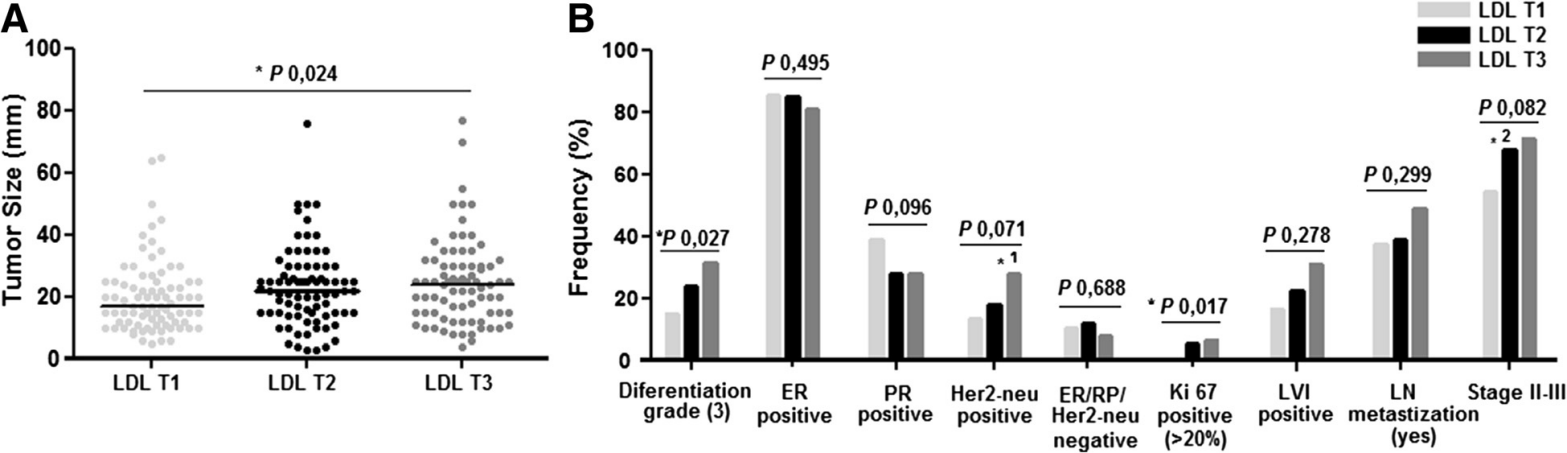
**Tumor characteristics in LDL-C tertiles groups. A**. Tumor size increases across LDL-C tertiles groups. Line represents the median value of tumor size in each LDL-C tertile. **B**. Frequency of tumor characteristics in LDL-C tertiles groups. ^*1^LDL T1-T2 (Her2-neu positive15,5%) vs LDL T3 (Her2-neu positive 27,8%): OR 5,015 (1,678-14,988) *P* 0,002. ^*2^LDL T1 (Stage II-III 54,9%) vs LDL T2-T3 (Stage II-III 69,6%): OR 0,543 (0,313-0,943) *P* 0,029. Kruskall-Wallis test. ER: Estrogen Receptor; PR: Progesterone Receptor; LVI: Lymphovascular Invasion; LN: Lymph Nodes.

Population was stratified based on LDL-C level tertiles: LDL T1:LDL-C ≤ 117 mg/dl; LDL T2:144 mg/dl ≥ LDL-C > 117 mg/dl; LDL T3:LDL-C > 144 mg/dl. Patients in the third LDL-C tertile have larger tumors (*P* 0,024) (Figure 1A), of higher differentiation grade (*P* 0,027), with higher proliferative rate (*P* 0,017), and are diagnosed in more advanced stages. This analysis does not demonstrate differences in lipid profiles between breast tumor immunohistochemical subtypes (Luminal A, Luminal B, Triple negative and HER2 type [61]). Nevertheless, tumors of patients in the third LDL-C tertile are more commonly Her2-neu positive, when compared to others tertiles (*P* 0,002) (Figure 1B). There are no differences, between LDL-C categories, concerning studied BC risk factors (Additional file 5).

### Multivariate logistic regression

A multivariate logistic regression to the risk of tumor T stage was then modeled. All the variables significantly associated at univariate analysis (Additional file 6) were introduced. Age and BMI, even not positively associated, were also included because of its strong correlation with lipid profile. It was found that the LDL-C level > is a predictor factor to tumor size ≥ 20 mm, at diagnosis (Table 2).

**Table 2:** Univariate and multivariate logistic regression to the risk of tumor size ≥20 mm

	***Univariate analysis***			***Multivariate analysis***		
**Variable**	***HR***	***95% CI***	***P value***	***HR***	***95% CI***	***P value***
**Total cholesterol** T≥2vs T1	1,912	1,113-3,285	0,018			
**LDL-C (>117mg/dl)** T≥2vs T1	2,419	1,394-4,199	0,002	2,468	1,356-4,491	0,003
**Triglycerides** T3	1,888	1,092-3,264	0,022			
**BMI** T≥2vs T1	1,785	0,010-3,155	0,045			
**Age** T≥2vs T1	0,833	0,430-1,416	0,499			

LDL-C: Low Density Lipoprotein; BMI: body mass index; HR: hazard ratio, CI: confidence interval.

### Survival and cox regression model

**Figure 2:**
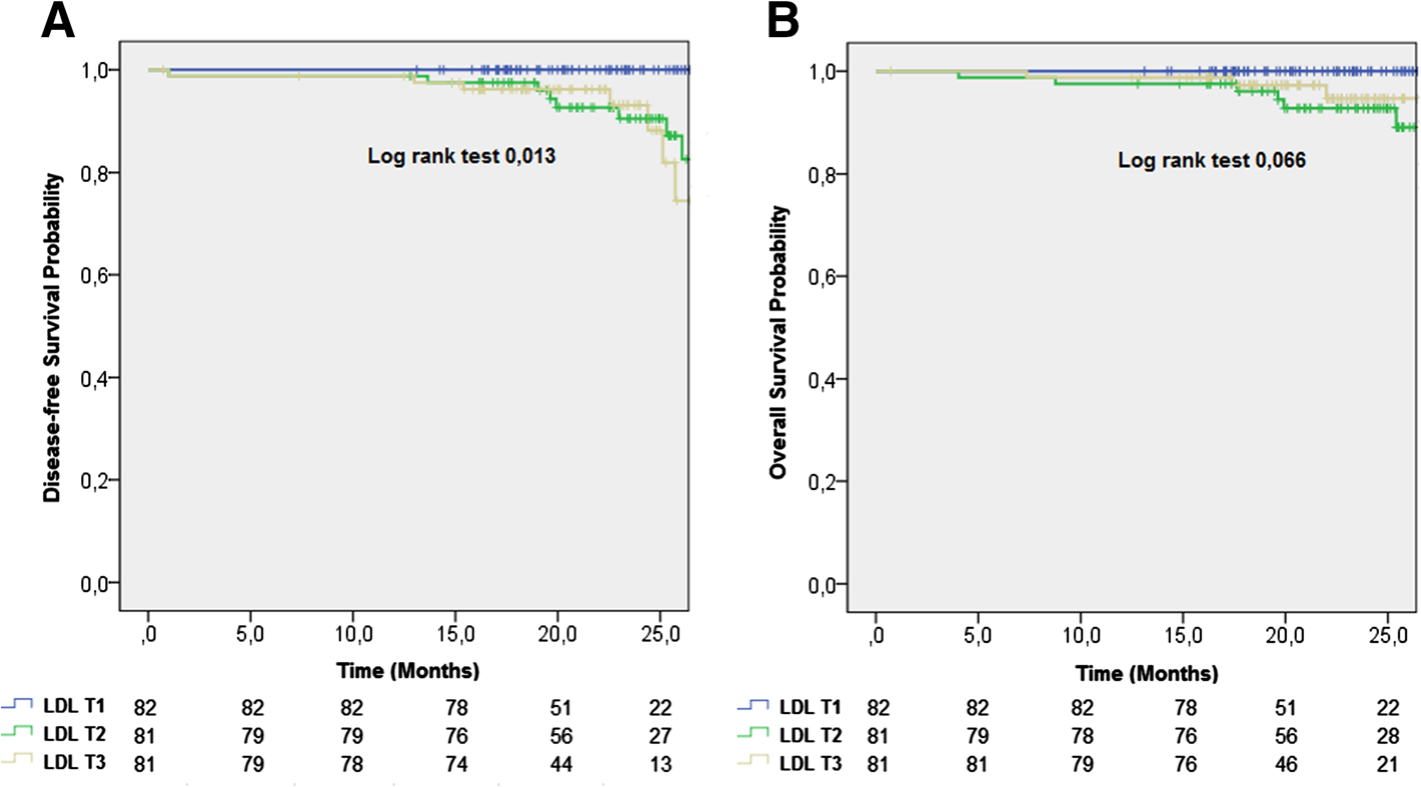
**Overall and disease-free survival in LDL-C tertiles groups.** Kaplan–Meier Curves. **A**. At 25 months, overall survival is 100% in LDL T1, 92,8% in LDL T2 and 97,2% in LDL T3 (Log rank test *P* 0,066). **B**. At 25 months, disease-free survival is 100% in LDL T1, 90,6% in LDL T2 and 88,3% in LDL T3 (Log rank test 0,013).

At 25 months of follow up the DFS in LDL T1, LDL T2 and LDL T3 groups was 100%, 90,6% and 88,3%, respectively (log rank test 0,013) (Figure 2A). OS had no statistically significant differences between LDL-C tertiles groups (Figure 2B).

Cox regression model to DFS, adjusted to tumor T and N stages, and BC immunohistochemical subtypes, revealed that LDL-C higher than 117, mg/dL, at diagnosis, is associated with poor disease-free survival (HR 0,129; CI 0,017-0, 978, *P* 0,048) (Table 3).

**Table 3:** Cox multivariate regression model for disease-free survival

**Variable**	***HR***	***95% CI***	***P value***
**Tumor T stage** (≥T2)	0,822	0,264-2,565	0,736
**Tumor N stage** (≥N1)	0,551	0,201-1,515	0,248
**LDL-C** (> 117 mg/dL)	0,129	0,017-0,978	0,048
**Luminal A** (yes)	0,599	0,072-5,017	0,637
**Luminal B** (yes)	0,532	0,047-6,026	0,610
**Triple negative** (yes)	0,128	0,015-1,111	0,062
**HER2 type** (yes)^a^	–	–	–

^a^Degree of freedom reduced because of constant or linearly dependent covariates.

LDL-C: Low Density Lipoprotein; T and N: TNM stages; HR: hazard ratio, CI: confidence interval.

## Discussion

Multiple epidemiological studies exploring causal associations between dyslipidemia and BC incidence produced contradictory results [24-37]. Several methodological aspects may explain the diverse conclusions, but the influence of cholesterol in BC risk remains to be clinical demonstrated.

Nevertheless, biological clues from laboratory [13-19] and *in vivo* pre-clinical studies [20-23], as well as significant alterations in lipid profile of BC patients compared to healthy controls [41-49],56,[57],62,63], are very suggestive for a role of cholesterol in BC progression.

In the present study, fasting lipid profile (with discrimination between lipids and lipoproteins fractions) was prospectively assessed in a cohort of patients with invasive carcinoma, in initial stages, before any treatment and with no history of being on antidiabetic or lipid lowering drugs (including statins, fibrates, oral antidiabetics, insulin or corticosteroids). Population characteristics are similar to other series, concerning to demographic and tumor characteristics [64-66]. A slight under-representation of triple negative and HER2 type cancer have occurred due to the inclusion criteria, as those patients are more likely to undergo neoadjuvant chemotherapy. The average lipid profile of BC patients in this cohort superimposes that of the sex, age and BMI-matched portuguese population [67,68].

Results show that systemic LDL-C level above 117 mg/dL is a predictive factor of tumor T stage, at diagnosis. These levels are also positively associated with worse prognostic characteristics such as higher histological grade, higher proliferative rate [69] and more advanced clinical stage (II-III). Patients in the third tercile (LDL-C >144 mg/dl) are also more prone to have LVI and lymph node metastasis. This trend seems to be transversal to all immunohistochemical BC subtypes, although we found a significant higher number of Her2-neu positive cases in patients of the third tertile group.

Other published studies, also focused on assessing lipid profile in BC patients, found higher TC levels in BC patients, compared to healthy control patients [50,52] and also an association between increased TC, LDL-C and decreased HDL-C levels with increasing tumor stage [50].

To our knowledge this is the first cohort of BC patients in which the correlation of lipid profile and tumor characteristics was done in a setting of pre-treatment and with all patients free of lipid lowering drugs. Despite we did not accessed variables that may also influence the lipid profile such as smoking habits, type of diet, residence area or socioeconomic status, the most important co-variables, BMI, age and lipid lowering drugs were controlled.

Furthermore, we prospectively followed-up the patients, and at 25 months of follow up, overall survival did not evidenced differences but DFS of the patients in the third LDL-C tertile, at diagnosis was significantly reduced (88,3% vs 100%, *P* 0,013). Differences of DFS in the LDL tertiles groups, adjusted to BMI remains statistically significant (Additional file 7). Cox regression model shows that LDL-C level at diagnosis is inversely correlated with DFS, even considering tumor T and N stages and immunohistochemical subtypes covariates.

We found that LDL-C fraction is significantly associated with BC progression and may actually be useful in the identification and medical follow-up of high-risk groups. LDL-C levels at diagnosis therefore emerge as a prognostic factor in BC patients.

For operable BC, two years of follow up may be short, but is also reported that disease relapse has a peak of incidence in the first two years after diagnosis [70,71]. As a limitation we could not avoid the possible influence of adjuvant treatment either in lipid profile or disease progression, once no modification to the routine protocols was introduced. Nevertheless the strong association of LDL-C level and tumor size before treatment favors the LDL-C as a putative prognostic biomarker. It is also not possible to exclude the common association of cholesterol levels, obesity and the variance in health awareness before diagnosis. However results were adjusted to BMI and, during follow up, all women were evolved in the same program of surveillance and health control.

We can speculate that high LDL-C levels in patients with aggressive (high stage/ high grade) primary tumors have a cancer-fuelling effect and are a co-causative factor, in patients with chronic hypercholesterolemia. But, on the other hand, the high LDL-C levels that we observed may actually reflect a shift in cholesterol synthesis (by the liver or tumor cells themselves) in patients with aggressive tumors; accordingly, Zielinski et al. [72] followed-up a group of patients with advanced BC in remission and described a significant rise in plasma cholesterol and triglycerides in most of those who developed disease progression.

Considering that proliferating cancer cells have an increased demanding of cholesterol and intermediates of cholesterol biosynthesis pathway, the up-regulation of cholesterol biosynthesis and reduced cellular efflux are expected. In cancer cells, cholesterol synthesis has been shown to be increased, due to availability of precursors or to increased transcription [73], and this may have contributed to BC carcinogenesis [74,75]. Hidroxi-3-methyl-glutaril-coA reductase 3 inhibition by statins decreases *in vitro* cell proliferation, attesting that cholesterol biosynthesis should be important to tumor growth [74]. Moreover, elevated cholesterol content is characteristic of breast tumors [76] and acyl-CoA: cholesterol acyltransferase 1 inhibition, an enzyme involved in cholesteryl esterification decreases proliferation and invasion rate [13].

However, despite cancer cells increases intracellular cholesterol synthesis, this effect is not expected to produce hypercholesterolemia and justify the observed associations.

Some other published data supports the notion that cancer cells are able to uptake cholesterol from the bloodstream. High LDL-C receptor expression was demonstrated in BC tissue, compared to normal tissue [77]. So plasma LDL-C could be used by cancer cells. In *in vitro* experiments, was also demonstrated that cancer cells can also consume HDL-C through the scavenger receptor class B, type I (SR-BI) [15,16,78] and exogenous triglycerides [79]. In our clinical setting HDL-C or triglycerides measurements did not show suspicious modifications, but our results do not rule out the possibility of cancer cells consume.

Corroborating this hypothesis are laboratory studies showing that both exogenous LDL-C and HDL-C can promote proliferation and migration, features of aggressive tumors [14-16]. Also animal studies showed a higher tumor and metastatic burden [21] as well as disease progression [22,23] in hypercholesterolemic mice compared with non- hypercholesterolemic controls.

The exogenous cholesterol could be mobilized from body storage, through HDL-C or from diet, through hepatic metabolism and LDL-C. Mobilization is not likely to contribute to hyperlipidemia, since we saw the same pattern of lipid profile in BC patients and in the age and sex-matched non-cancer portuguese population [67,68].

High LDL-C and low HDL-C is the most common lipid profile induced by western diet and is highly frequent [80].

Initial studies on cholesterol and cancer showed an increased risk in patients with lower plasma cholesterol. Once demonstrated that reduction in cholesterol level does not cause cancer this has been assumed as a preclinical effect of malignancy considering that cancer cells up take cholesterol and decreases its levels. [62,63]. We found an association of LDL-C with large tumors, but all tumors were at very initial clinical phase, many of them diagnosed in screening programs.

The causal relationship between systemic cholesterol has been hard to demonstrate because of the length of time to the event, multifactorial etiology of BC, concomitant medication, statistical analysis and event definitions. However, assuming that cholesterol is essential to cell proliferation, once a tumor develops in a hypercholesterolemic environment it is well adapted to progress and this may be the explanation for the observed association of LDL-C level, tumor size and disease progression.

We also found that tumors of the LDL-C highest tertile are more commonly Her2-neu (ErbB2) positive. Moreover, as mentioned earlier, membrane cholesterol is specially localized in the lipid rafts domains. Such areas are enriched in transmembrane molecules that are key in signaling pathways associated with malignant progression: Fas receptor, TNF related apoptosis-inducing ligand, AKT, integrins, cadherins and growth factor receptors [8] including ErbB2. The later is a tyrosine kinase receptor (and oncogene) localized in lipid rafts and its function is highly dependent on membrane fluidity [81,82]; cholesterol enrichment within the cell may therefore alter receptor-signaling. It is possible that ErbB2 receptor ligand-independent activation is potentiated by cholesterol-enriched environment, explaining a selection of Her2-neu positive tumors.

Although synthesis of estrogen and progesterone require cholesterol, no association of LDL-C and ER status was seen. *In vitro* and *in vivo* studies have showed contradictory results concerning the effect of cholesterol, in promoting ER positive breast cancer proliferation [14,20].

The pro-inflammatory microenvironment induced by high-cholesterol levels, as seen in atherosclerosis, in which LDL-C is the most important causative factor [83,84], can also play an effect on BC initiation and progression. The use of statins before cancer diagnosis reduces cancer related mortality [85]; the reduction of LDL-C is supposed to be the main mechanism through which statins exert effect, but the anti-inflammatory effect cannot be ruled out.

In our prospective study, patients with high levels of LDL-C at diagnosis had reduced disease free survival, even adjusting to tumor type, stage and BMI. This may reflect that high levels of LDL-C potentiate micrometatasis development or that hypercholesterolemia previously selected aggressive tumors but reinforces a role for systemic cholesterol in BC progression and cholesterol metabolism as a therapeutic target.

## Conclusion

We found that LDL-C fraction is significantly associated with BC progression and may actually be useful in the identification and follow-up of high-risk groups of BC patients. LDL-C levels at diagnosis, therefore emerge as a prognostic factor in BC. Additionally, results support a role for systemic cholesterol in BC progression and indicate that cholesterol metabolism could be a therapeutic target in BC treatment.

## Competing interest

The authors have no financial or non-financial competing interests.

## Authors’ contribution

**CRS**: Study design, patient enrollment, data collection and analysis, manuscript writing. **IF**: Pathological data validation and analysis, manuscript revision. **SD**: Study design, data analysis, manuscript writing. **MA**: Study supervision, data analysis, manuscript revision. All authors read and approved the final manuscript.

## Pre-publication history

The pre-publication history for this paper can be accessed here:

http://www.biomedcentral.com/1471-2407/14/132/prepub

## Supplementary Material

Additional file 1 Breast Cancer Treatment (N=244). Click here for file

Additional file 2 Follow up (N=244). Click here for file

Additional file 3 Lipid Profile in Tumor Stage and in Prognostic Groups. Click here for file

Additional file 4 **Tumor Characteristics in BMI Tertiles Groups.** A. Tumor size across BMI tertiles groups does not show significant differences. Line represents the median value of tumor size in each BMI tertile. B. Frequency of tumor characteristics in BMI tertiles groups. Kruskall-Wallis test. ER: Estrogen Receptor; PR: Progesterone Receptor; LVI: Lymphovascular Invasion; LN: Lymph Nodes. Click here for file

Additional file 5 Patient Characteristics in LDL-C level tertiles. Click here for file

Additional file 6 Univariate Logistic Regression to the Risk of Tumor Size ≥20 mm. Click here for file

Additional file 7 **Overall and Disease-free Survival in LDL-C Tertiles Groups adjusted to BMI.** Kaplan–Meier Curves for overall survival and disease free survival to each BMI tertile. are shown. At 25 months of follow up disease-free survival shows differences across LDL-C tertiles, BMI adjusted (Log rank test *P* 0,018). But overall survival is not different in LDL-C tertiles (Log rank test 0,157). Click here for file
