# Supplementary material for: Plasma level of LDL-cholesterol at diagnosis is a predictor factor of breast tumor progression
Source: BMC Cancer. 2014 Feb 26;14:132. doi: 10.1186/1471-2407-14-132 (PMC3942620; doi:10.1186/1471-2407-14-132)
Supplement: Additional file 1 — Breast Cancer Treatment (N=244). [file 1471-2407-14-132-S1.doc]

| **Additional file 1. Breast Cancer Treatment (N=244)** | | | |
| --- | --- | --- | --- |
| **Treatment** |  | **No. of Patients** | **%** |
| **Surgery** |  |  |  |
| **Mastectomy** |  | 64 | 26,2 |
| **Breast Conserving** |  | 180 | 73,8 |
| **Chemotherapy** |  | 165 | 67,6 |
| **Trastuzumab** |  | 29* | 11,9 |
| **Radiotherapy** |  | 191 | 78,3 |
| **Endocrine Therapy** |  | 171 | 70,1 |
| * 13 cases Her2 Type; 16 cases Luminal B type | | | |
