# Supplementary material for: Plasma level of LDL-cholesterol at diagnosis is a predictor factor of breast tumor progression
Source: BMC Cancer. 2014 Feb 26;14:132. doi: 10.1186/1471-2407-14-132 (PMC3942620; doi:10.1186/1471-2407-14-132)
Supplement: Additional file 2 — Follow up (N=244). [file 1471-2407-14-132-S2.doc]

| **Additional file 2. Follow up (N=244)** | | | |
| --- | --- | --- | --- |
| **Follow up Status** |  | **No. of Patients** | **%** |
| **Follow up time** (months), median (interquartile range) |  | 22,3 (18,2-25,2) |  |
| **Folow up Status** (N,%) |  | 244 | 100 |
| **Local recurrence** |  | 1 | 0,4 |
| **Systemic disease progression** |  | 9 | 3,7 |
| **Death with disease** |  | 7 | 2,9 |
| **Death without disease** |  | 2 | 0,8 |
