# Supplementary material for: Plasma level of LDL-cholesterol at diagnosis is a predictor factor of breast tumor progression
Source: BMC Cancer. 2014 Feb 26;14:132. doi: 10.1186/1471-2407-14-132 (PMC3942620; doi:10.1186/1471-2407-14-132)
Supplement: Additional file 3 — Lipid Profile in Tumor Stage and in Prognostic Groups. [file 1471-2407-14-132-S3.doc]

| **Additional file 3. Lipid Profile in Tumor Stage ^1^ and in Prognostic Groups^2^** | | | | | | | |
| --- | --- | --- | --- | --- | --- | --- | --- |
| **Stage/ Level** (Median, interquartile range) | **Tumor Stage T1**  **(≤ 2cm)** | **Tumor Stage T2**  **(2-5cm)** | **Tumor Stage T3**  **(>5cm)** | ***P* value*^1^** | ***Stage I*** | ***Stage II-III*** | ***P* value*^2^** |
| **TC level, mg/dl** | 205  (183-228) | 212  (191-234) | 196  (189-225,5) | 0,105 | 202  (179,5-227) | 210  (194-233) | 0,053 |
| **HDL-C level, mg/dl** | 53  (48-62) | 52  (47-60) | 51,5  (45,5-53,8) | 0,229 | 53  (46-62) | 52,5  (46,3-60) | 0,566 |
| **LDL-C level, mg/dl** | 125  (100-148) | 131  (109,5-156) | 138  (118-159,5) | 0,015 | 124  (96,5-147) | 130,5  (114-153) | 0,013 |
| **TAG level, mg/dl** | 89  (75,5-117,5) | 104  ( 75-132) | 100  (72,5-115) | 0,212 | 92  (77,3-118,8) | 97  (74,5-129,5) | 0,512 |
| **LDL-C/**  **HDL-C** | 2,3  (1,7-3,1) | 2,6  (1,9-3,1) | 2,3  (2,4-2,8) | 0,020 | 2,3  (1,7-3,1) | 2,6  (2,0-3,1) | 0,040 |
| ^1^ TNM Classification. ^2^ The American Joint Committee on Cancer (AJCC) Staging System ^*1^ Kruskall-Wallis Test, ^*2^ Mann-Whitney Test. TC: total cholesterol; LDL-C: Low Density Lipoprotein; HDL-C: High Density Lipoprotein; TAG: triglycerides | | | | | | | |
