# Supplementary figures and images for: Plasma level of LDL-cholesterol at diagnosis is a predictor factor of breast tumor progression
Source: BMC Cancer. 2014 Feb 26;14:132. doi: 10.1186/1471-2407-14-132 (PMC3942620; doi:10.1186/1471-2407-14-132)

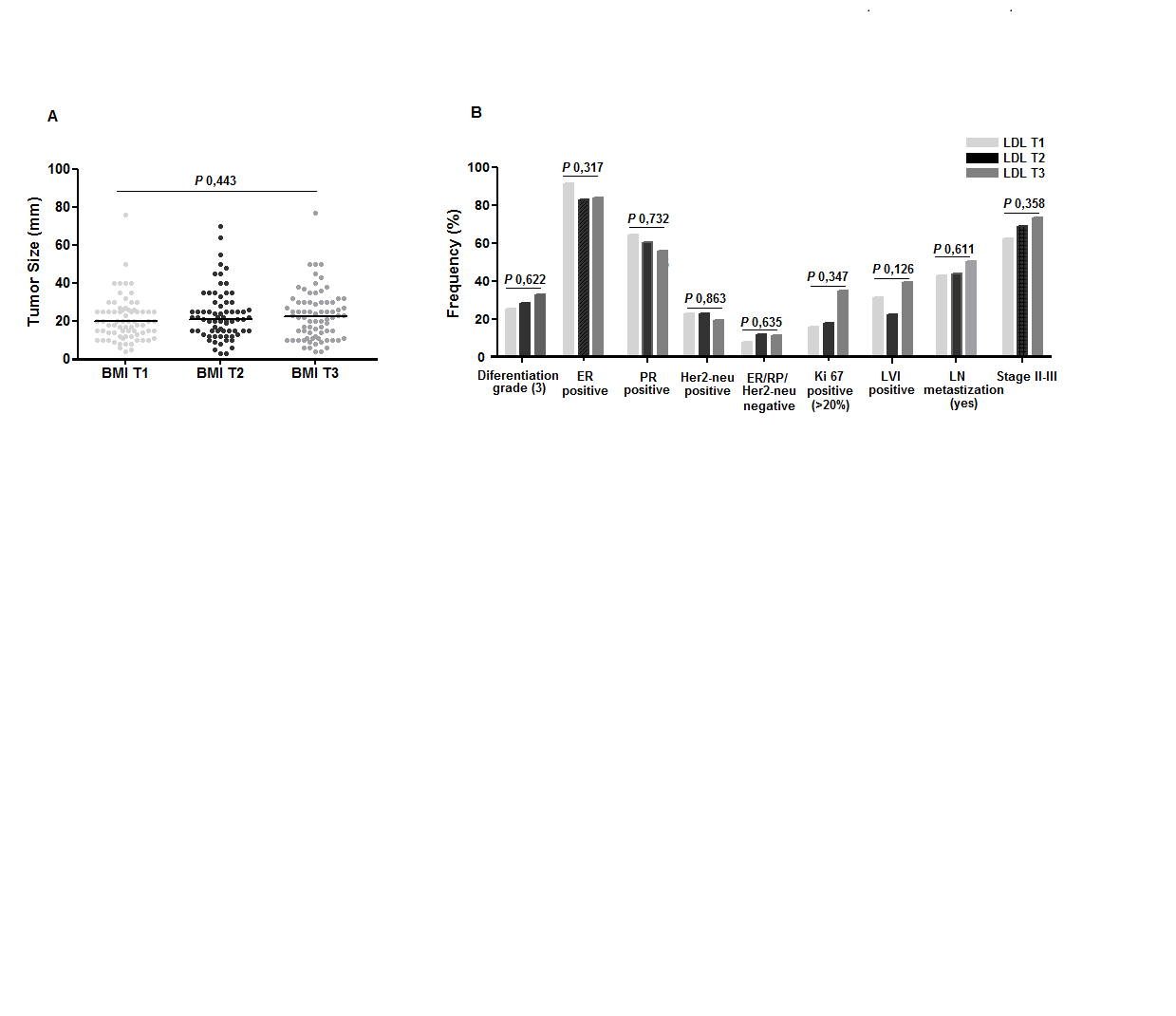

Supplement: Additional file 4 — Tumor Characteristics in BMI Tertiles Groups. A. Tumor size across BMI tertiles groups does not show significant differences. Line represents the median value of tumor size in each BMI tertile. B. Frequency of tumor characteristics in BMI tertiles groups. Kruskall-Wallis test. ER: Estrogen Receptor; PR: Progesterone Receptor; LVI: Lymphovascular Invasion; LN: Lymph Nodes. [file 1471-2407-14-132-S4.png]

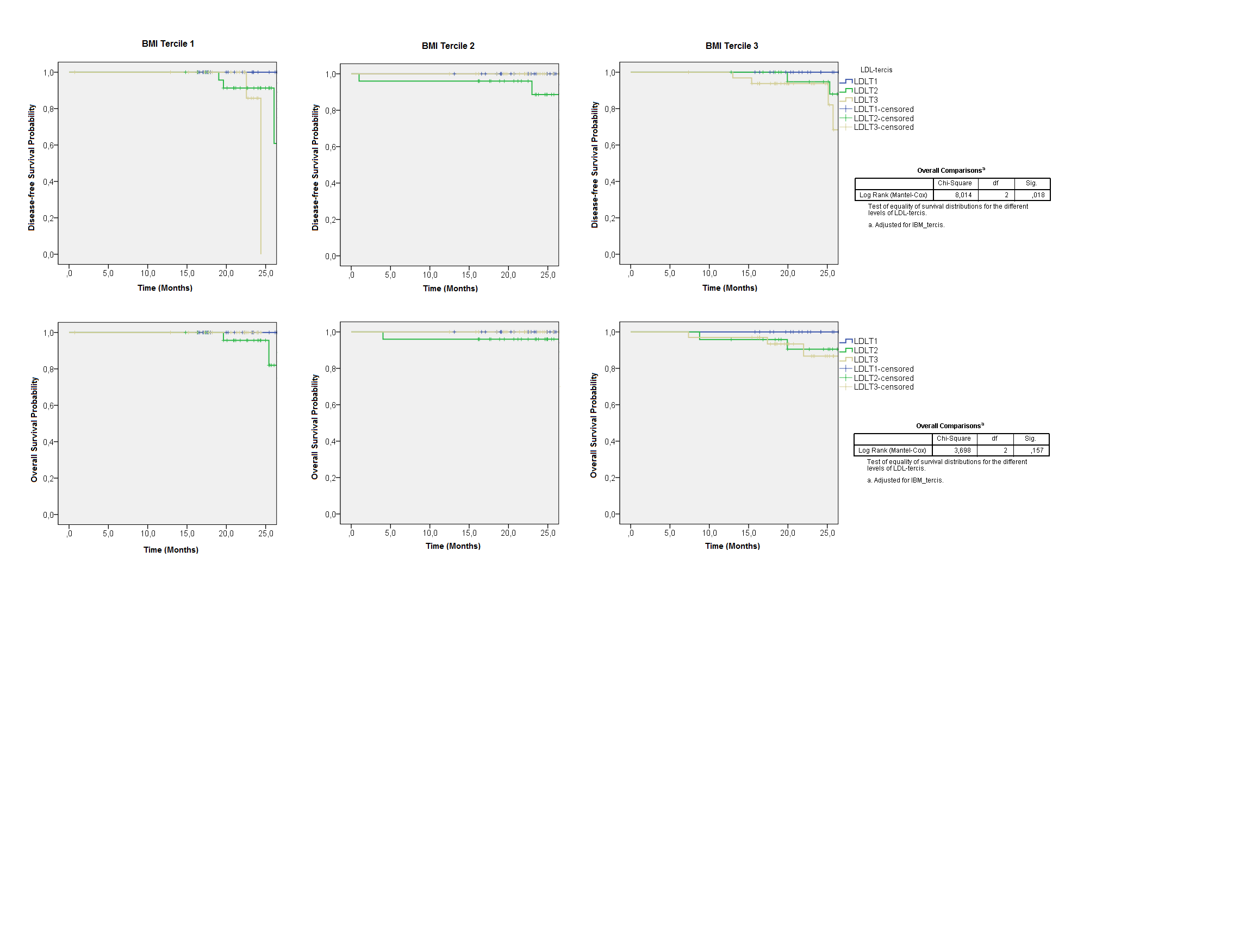

Supplement: Additional file 7 — Overall and Disease-free Survival in LDL-C Tertiles Groups adjusted to BMI. Kaplan–Meier Curves for overall survival and disease free survival to each BMI tertile. are shown. At 25 months of follow up disease-free survival shows differences across LDL-C tertiles, BMI adjusted (Log rank test P 0,018). But overall survival is not different in LDL-C tertiles (Log rank test 0,157). [file 1471-2407-14-132-S7.png]
