# Supplementary material for: Plasma level of LDL-cholesterol at diagnosis is a predictor factor of breast tumor progression
Source: BMC Cancer. 2014 Feb 26;14:132. doi: 10.1186/1471-2407-14-132 (PMC3942620; doi:10.1186/1471-2407-14-132)
Supplement: Additional file 5 — Patient Characteristics in LDL-C level tertiles. [file 1471-2407-14-132-S5.doc]

| **Additional file 4: Patient Characteristics in LDL-C levels tertiles** | | |  |  |
| --- | --- | --- | --- | --- |
| **Patient**  **Characteristic** | **LDL T1**  **(LDL≤117mg/dl)**  **N=82** | **LDL T2**  **(144mg/dl≥LDL>117mg/dl)**  **N=81** | **LDL T3**  **(LDL>144mg/dl)**  **N= 81** | ***P* value** |
| **Age (years)**, median (interquartile range) | 60,14 (49,0-70,3) | 58,3 (48,2-70,0) | 58,2(50,9-64,2) | 0,593 |
| **Menopausal Status (+),** (N, %) | 64,7% | 65,1% | 67,2% | 0,951 |
| **Pregnancy History (+)**, (N, %) | 94,2% | 71,9% | 88,3% | 0,408 |
| **Breast –feeding (+)**, (N,%) | 76,6% | 51,9% | 68,4% | 0,079 |
| **Oral contraception** (+), (N,%)  **/ HT (+)** | 51,6% | 58,3% | 53,7% | 0,597 |
| **Family History♯** | 40% | 34,5% | 25,5% | 0,258 |
| **BMI(Kg/m^2^**), median (interquartile range) | 25,7(22,8-29,4) | 26,1(23,4-29,4) | 27,9(24,7-31,5) | 0,107 |
| HT: Hormonal Therapy; BC: Breast Cancer; BMI: Body Mass Index, ♯ not in the first generation. *P* value: Kruskall-Wallis test. LDL-C: Low Density Lipoprotein; BMI: body mass index; T: tertile level. | | | | |
