# Supplementary material for: Plasma level of LDL-cholesterol at diagnosis is a predictor factor of breast tumor progression
Source: BMC Cancer. 2014 Feb 26;14:132. doi: 10.1186/1471-2407-14-132 (PMC3942620; doi:10.1186/1471-2407-14-132)
Supplement: Additional file 6 — Univariate Logistic Regression to the Risk of Tumor Size ≥20 mm. [file 1471-2407-14-132-S6.doc]

| **Additional file 5. Univariate Logistic Regression to the Risk of Tumor Size ≥20mm** | | | | |
| --- | --- | --- | --- | --- |
| **Variable** | ***Univariate Analysis***  ***HR 95% CI P value*** | | | **N** |
| **Total Cholesterol**  T1 | 0,491 | 0,285-0,845 | 0,010 | 244 |
| **Total Cholesterol**  T≥2 T1vsT2.T3 | 1,912 | 1,113-3,285 | 0,018 | 244 |
| **Total Cholesterol**  T3 | 1,489 | 0,869-2,550 | 0,146 | 244 |
| **Total Cholesterol**  Q1 | 0,555 | 0,312-0,070 | 0,081 | 244 |
| **Total Cholesterol**  Q4 | 1,351 | 0,763-2,395 | 0,301 | 244 |
| **HDL-C** T1 | 1,458 | 0,849-2,504 | 0,171 | 241 |
| **HDL-C** T≥2 | 0,686 | 0,399-1,178 | 0,171 | 241 |
| **HDL-C** T3 | 0,739 | 0,430-1,268 | 0,271 | 241 |
| **HDL-C** Q1 | 1,231 | 0,690-2,195 | 0,482 | 241 |
| **HDL-C** Q4 | 0,686 | 0,390-1,207 | 0,190 | 241 |
| **LDL-C** T1 | 0,413 | 0,238-0,718 | 0,002 | 243 |
| **LDL-C**  T≥2 | 2,419 | 1,394-4,199 | 0,002 | 243 |
| **LDL-C** T3 | 1,556 | 0,904-2,677 | 0,133 | 243 |
| **LDL-C** Q1 | 0,305 | 0,167-0,557 | <0,0001 | 243 |
| **LDL-C** Q4 | 1,709 | 0,952-3,069 | 0,071 | 243 |
| **Triglycerides** T1 | 0,951 | 0,555-1,629 | 0,855 | 241 |
| **Triglycerides**  T≥2 | 1,051 | 0,614-1,800 | 0,855 | 241 |
| **Triglycerides** T3 | 1,888 | 1,092-3,264 | 0,022 | 241 |
| **Triglycerides** Q1 | 0,989 | 0,552-1,772 | 0,970 | 241 |
| **Triglycerides** Q4 | 1,628 | 0,898-2,953 | 0,107 | 241 |
| **BMI** T1 | 0,560 | 0,117-0,990 | 0,045 | 221 |
| **BMI** T≥2 | 1,785 | 1,010-3,155 | 0,045 | 221 |
| **BMI** T3 | 1,438 | 0,823-2,512 | 0,201 | 221 |
| **Age** T1 | 1,453 | 0,848-2,487 | 0,173 | 244 |
| **Age** T≥2 | 0,688 | 0,402-1,179 | 0,173 | 244 |
| **Age** T3 | 0,833 | 0,430-1,416 | 0,499 | 244 |
| LDL-C: Low Density Lipoprotein; HDL-C: High Density Lipoprotein, BMI: Body Mass Index, T: tertile level, Q: quartile level; CI: Confidence Interval. | | | | |
